# Current Limitations of Surgical Robotics in Reconstructive Plastic Microsurgery

**DOI:** 10.3389/fsurg.2018.00022

**Published:** 2018-03-22

**Authors:** Youri P. A. Tan, Philippe Liverneaux, Jason K. F. Wong

**Affiliations:** ^1^The Manchester Centre for Plastic Surgery and Burns, Manchester University NHS Foundation Trust, Manchester, United Kingdom; ^2^Department of Hand Surgery, University Hospital of Strasbourg, FMTS, University of Strasbourg, Icube CNRS, Illkirch, France; ^3^Blond McIndoe Laboratories, Division of Cell Matrix Biology and Regenerative Medicine, University of Manchester, Manchester, United Kingdom

**Keywords:** reconstructive surgery, plastic surgery, microsurgery, surgical robotics, robotic surgery

## Abstract

Surgical robots have the potential to provide surgeons with increased capabilities, such as removing physiologic tremor, scaling motion and increasing manual dexterity. Several surgical specialties have subsequently integrated robotic surgery into common clinical practice. Plastic and reconstructive microsurgical procedures have not yet  benefitted significantly from technical developments observed over the last two decades. Several studies have successfully demonstrated the feasibility of utilising surgical robots in plastic surgery procedures, yet limited work has been done to identify and analyse current barriers that have prevented wide-scale adaptation of surgical robots for microsurgery. Therefore, a systematic review using PubMed, MEDLINE, Embase and Web of Science databases was performed, in order to evaluate current state of surgical robotics within the field of reconstructive microsurgery and their limitations. Despite the theoretical potential of surgical robots, current commercially available robotic systems are suboptimal for plastic or reconstructive microsurgery. Absence of bespoke microsurgical instruments, increases in operating time, and high costs associated with robotic-assisted provide a barrier to using such systems effectively for reconstructive microsurgery. Consequently, surgical robots provide currently little overall advantage over conventional microsurgery. Nevertheless, if current barriers can be addressed and systems are specifically designed for microsurgery, surgical robots may have the potential of meaningful impact on clinical outcomes within  this surgical subspeciality.

## 1. Introduction

**Figure 1:**
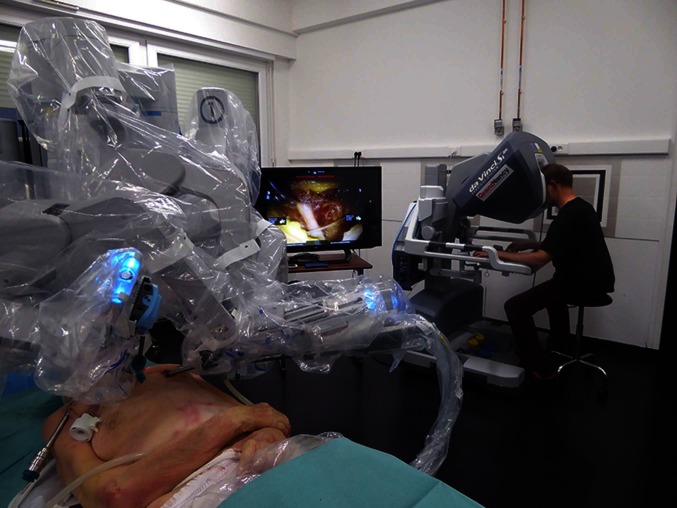
Endoscopic robot-assisted C7 nerve root retrophalangeal transfer from the contralateral healthy side: a cadaver feasibility study. Installation of a da Vinci SI^®^ robot (Intuitive Surgical^™^, Sunnyvale, CA). In the first plane, the cadaver is in a supine position and the robot is installed at the head to dissect the left brachial plexus. In the second plane, a 2D monitor shows the suture of the two current C7 roots. In the third plane, the operator, installed at the surgical console, telemanipulates the robot's arms. The working space is kept open by blowing CO2 at 10 mmHg.

Progressive changes in robotic and computer-guided systems have significantly altered operating practice across different medical and surgical specialties. Surgical robots can extend the capabilities of surgeons by reducing fine tremors, increasing manual dexterity and offering real-time 3D visualisation during endoscopic surgery (1, 2). This allows for highly precise  movement in narrow and difficult-to-access spaces, and subsequently opens the door for minimally invasive surgery (Figure 1). In addition to enhancing surgical skills and improving ergonomics during surgery, minimally invasive surgery is associated with shorter hospitalisation and reduced risk of adverse complications (3, 4). Recent success of surgical robots, particularly the da Vinci Surgical System^®^ (Intuitive Surgical^™^, Sunnyvale, CA), has created a thriving multibillion-dollar industry. Numerous companies are now focusing on this market in order to gain a stake in this valuable industry. Verily Life Sciences^™^ (formerly Google Life Sciences^™^) and Johnson & Johnson^™^ are collaborating on the development of a surgical robotic system through Verb Surgical^™^. Medtronic^™^ has made sizeable investments in Mazor Robotics^™^ that focusses on the development of surgical robots for spine and brain surgery. Furthermore, other less-known companies, such as Auris Surgical Robotics^™^, Cambridge Medical Robotics^™^ and TransEnterix Surgical^™^, have each announced their own surgical robotics platform. While several novel teleoperated surgical robotic systems are expected to become available within the foreseeable future, the next generation of partial autonomous surgical robots, capable of suturing skin and creating soft-tissue anastomosis without direct input from surgeons, is already being tested in research laboratories (5).

### 1.1. Surgical Robotics in Plastic Surgery

Despite numerous technical advancements in surgical robotics in recent years, only a small proportion of surgical specialties have benefited notably from these innovations. Within the United States, approximately 85% of radical prostatectomies were done robotically in 2013. Radical prostatectomies are now more commonly performed using a robot-assisted technique compared to laparoscopic surgery (6, 7). Similarly, uptake of robotic-assisted surgery has been increasing rapidly among gynaecology within a relatively short time frame. A survey among gynaecologist oncology training centres revealed that 24% of US gynaecologist oncologists perform robotic-assisted surgery, whereas 95% of US gynaecological oncology fellows are trained to use the da Vinci Surgical System^®^ (8).

 Contrary to the developments within urological and gynaecological surgery, the field of plastic and reconstructive surgery has seen little utilisation of surgical robotics and computer-assisted systems in the operating theatre. Despite plastic surgery’s limited use of endoscopic surgery, specific advantages of currently available robotic systems, such as removal of physiologic tremor and improved dexterity, can theoretically provide significant aid during microsurgical procedures. Motivated by the possibility of enhancing the practical skills required during surgery and combining this with imaging modalities, plastic surgeons have studied the feasibility of integrating robots into clinical practice. Several clinical studies have shown that robotic microsurgery for plastic and reconstructive procedures is feasible. Early studies in rats highlighted that robotic-assisted surgery can aid during microsurgery. In 2000, Li et al. successfully demonstrated 1 mm femoral artery anastomosis in Sprague-Dawley rats using a four-degrees-of-freedom telemanipulator system (SRI International, Menlo Park, CA) with equal anastomotic patency rates compared to conventional microsurgical anastomosis (9). A larger comparison in 2005 between conventional microsurgery and microsurgery using the Zeus Robotic Surgical System^®^ (Computer Motion^™^, Goleta, CA) to perform end-to-end anastomoses of 1 mm femoral arteries in Sprague-Dawley rats with interrupted 10–0 suture achieved similar results (10). Later studies in pigs also revealed positive results in more complex scenarios, such as free-flap surgery and limb replantation (11, 12). Success in a variety of animal studies, subsequently translated into the first human trials of robotic-assisted surgery to harvest the internal mammary vessels to provide the recipient pedicle for free-flap breast reconstruction. Granting a relatively high complication rate in 20 patients (6 take-backs, and loss of 2 muscle flaps), Boyd et al. highlighted that robotic harvest of the internal mammary vessels was feasible (13). As surgical robots became more  available in hospitals, they were applied to a wider range of plastic surgical procedures, such as free-flap reconstruction (14, 15), brachial plexus (16, 17) and peripheral nerve surgery (18). Yet, despite the theoretical benefits positive results, plastic surgery did not follow the trend to incorporate surgical robots on a larger scale. Adaptation of surgical robots within plastic surgery is currently limited to very few reconstructive microsurgical procedures. Sole subspecialty within plastic surgery that has utilised robotic-assisted surgery is oropharyngeal surgery, on the grounds that the da Vinci Surgical System^®^ is FDA approved for transoral robotic head and neck surgery. Nevertheless, using surgical robots during plastic surgery procedures remains a rare occurrence.

In order to maximise the potential of surgical robots in plastic and reconstructive surgery, barriers and shortcomings of surgical robots have to be addressed. In this paper, we examine previous studies and identify limitations of surgical robots and indirect barriers that have prevented widespread utilisation.This will help us determine what are the requirementsfor future robotic systems to address the needs in reconstructive microsurgery. The aim of our systematic review is to increase awareness of the imperfections of the current generation of surgical robots and prevent these from occurring in future robotic systems.

## 2. Methodology

A literature search was performed using MEDLINE, PubMed, Embase and Web of Science. The search term “robot* microsurgery” wasused for all four databases: MEDLINE, Embase, PubMed and Web of Science. Our search was limited to full-text articles written in English and published between January 2000 and July 2017. Only papers that directly utilised surgical robots in either a laboratory or clinical settings were included. Emphasis was put on feasibility studies, case reports, case series and so on, whereas literature reviews were excluded. Both human and animal studies were included in our search. For each paper, abstracts were assessed based on relevance to robotics within plastic reconstructive microsurgery. Publications purely related to other surgical specialties, such as urology and neurosurgery were excluded. In addition, engineering or technical papers proposing novel robotic systems were also omitted. Articles that passed initial screening were subsequently reviewed full text and data regarding limitations, disadvantages and barriers of surgical robotics in plastic and reconstructive microsurgery was extracted.

## 3. Results

**Figure 2:**
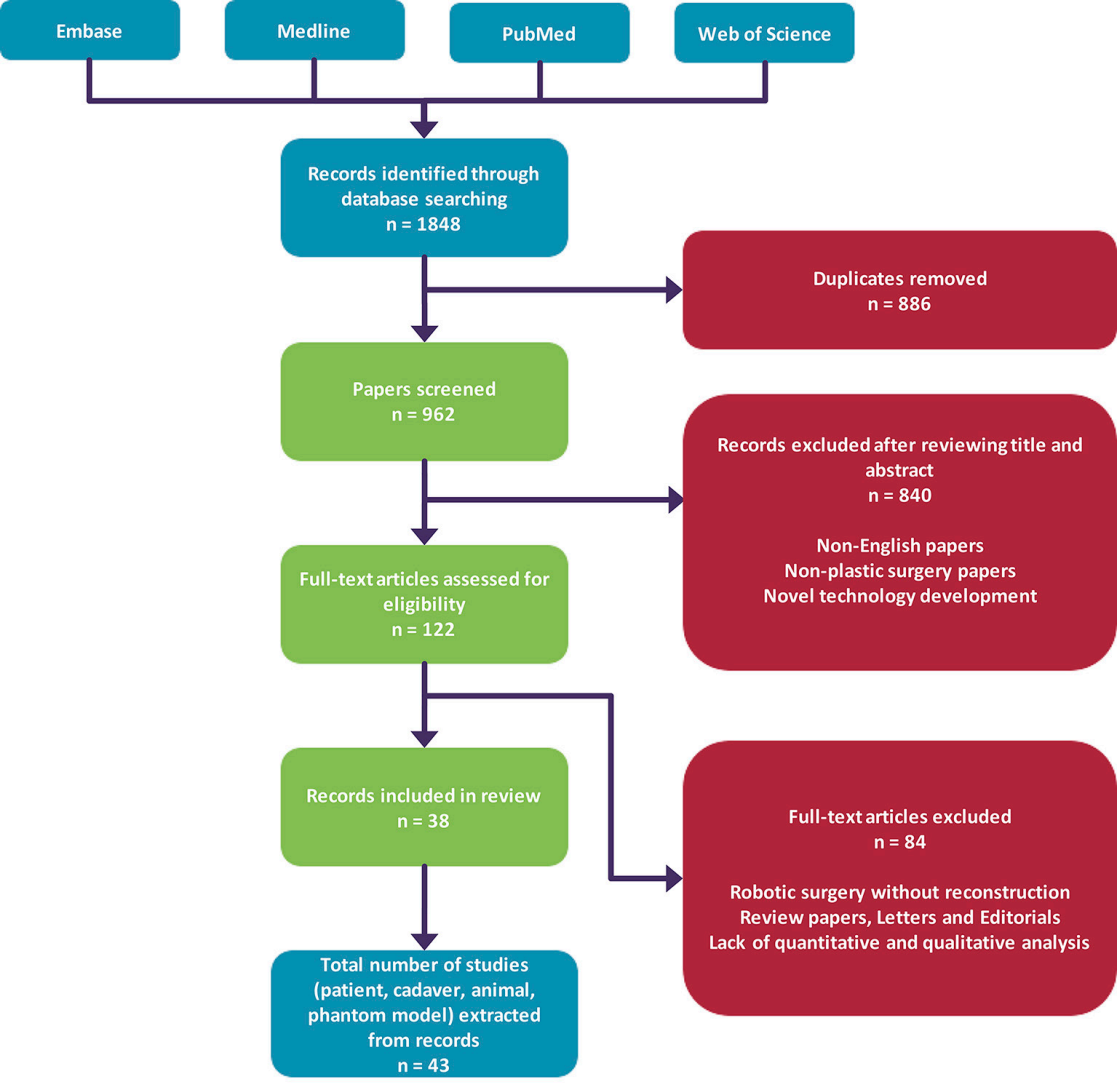
Flowchart of the database search strategy used in this review.

Our search yielded a total of 1,848 articles, and after removal of duplicates we obtained 962 unique articles. Eight hundred and forty articles were subsequently excluded after screening of abstracts based on our inclusion criteria as described in our methodology. Hundred and twenty-two full-text papers were assessed for further analysis, which resulted in exclusion of 84 articles and inclusion of 38 papers (Figure 2). Analysis of all papers in our review revealed that the 53% (*n* = 20) of papers that utilised surgical robotic systems during microsurgical procedures are feasibility studies. The remainder of papers were case series (*n* = 10), case reports (*n* = 4) and cohort studies (*n* = 2). The da Vinci Surgical System^®^ was the most common surgical robotic system that was used (*n* = 31). Other papers either used the Zeus Robotic Surgical System^®^, Automated Endoscopic System for Optical Positioning^®^ (Computer Motion^™^, Goleta, CA) or a custom surgical robot (Table 1). 

**Table 1:** Limitations of surgical robotics.

	Number of papers	Percentage
Total number of papers	38	100
**SURGICAL ROBOT**		
da Vinci Surgical System^®^	31	81.6
Zeus Robotic Surgical System^®^	3	7.9
Automated Endoscopic System for Optical Positioning^®^	1	2.6
Other	3	7.9
**DISADVANTAGES**		
Inadequate instrumentation	17	44.7
Increase in operating time	17	44.7
Absence of tactile feedback	16	42.1
High cost	13	34.2
Space requirements	10	26.3

Fourteen papers examined the use of surgical robotics in live humans patients (13–26), five studies exclusively focussed on human cadaver specimens (27–31), while two papers described the use of surgical robots in both human cadavers and live patients (32, 33). Fourteen papers evaluated the use of surgical robotics exclusively in animal models (9–12, 34–43), and one paper used both animal models and human cadaver material (44). A relatively small proportion of papers (*n* = 2) that were included in our review used artificial material to investigate the use of surgical robotic systems in reconstructive microsurgery (45, 46). Nearly all studies, 93% (*n* = 13) that described the use of surgical robots in live human patients utilised the da Vinci Surgical System^®^. The exact versions of the da Vinci Surgical System^®^ used across the different studies, for example, da Vinci S^®^, da Vinci Si^®^, da Vinci Xi^®^, could not be determined due to this specific data being omitted from a number of papers that were included in our review. Only one single study did not use the da Vinci Surgical System^®^ in patients, instead the Automated Endoscopic System for Optical Positioning^®^ was used. A total of 114 patients were operated on with the assistance of surgical robotics. Despite accounting for only 35% of studies (14, 15, 20, 22, 23, 25, 32), head and neck surgery accounted for 51% (*n* = 58) of patients that were operated on with the aid of surgical robotics. Reconstruction of the oropharynx following malignancy was the most common indication for transoral robotic-assisted surgery. Among this cohort, 40 patients underwent oropharyngeal reconstruction using a free radial forearm fasciocutaneous flap (14, 15, 20, 23, 25, 32), 4 patients were reconstructed using an anterolateral thigh flap (14, 20, 23), 1 patient benefitted from a facial artery myomucosal flap (23) and 1 patient had their defect repaired via primary closure (23). Apart from oropharyngeal malignancy, 10 patients underwent transoral robotic-assisted surgery for cleft palate repair (22) and 2 patients had scalp defects repaired using a free latissimus dorsi muscle flap (32). Breast surgery was the second most common surgical procedure during which surgical robotic systems were used (*n* = 26) (13, 32). Twenty patients had internal mammary vessels harvested using the Automated Endoscopic System for Optical Positioning^®^ for breast reconstruction (13). Six patients underwent robotic harvest of the latissimus dorsi muscle for free muscle transfer reconstruction of the breast (32). Furthermore, 12 patients underwent robot-assisted reconstructive upper-limb surgery during (18, 19, 21, 24), 7 patients received surgery for lower limb defects (24, 26, 33), 6 patients were operated on for pelvic reconstruction (26) and 5 patients had brachial plexus surgery (16, 17, 24) (Table 2). 

Analysis of any disadvantages or drawbacks of using surgical robotics for plastic reconstructive microsurgery, revealed five common obstacles (Table 1). Seventeen papers highlighted that the use of surgical robotics was associated with an increase in operating room time. Factors contributing to the increase in time were additional time necessary for setting up the surgical robot before a procedure (12, 15, 16, 36) and the additional time required to perform then actual procedure. Li et al. noted that total completion time was 2.6 times longer using a robotic-assisted technique compared to conventional microsurgery (9). Similarly, cleft palate repair by Nguyen et al., showed that the mean surgical duration for transoral robotic-assisted surgery was 122 ± 8 min, compared to 87 ± 6 min for the control group (22). An increase in operating time can partially be accounted to difficulties with surgical tools. Seventeenpapers mentioned that the current tools available in current robotic systems are inadequate for reconstructive microsurgical procedures. The lack of true microsurgical instruments creates a barrier for surgeons to handle delicate equipment and tissue. Consequently, needle tearing and difficulties in manipulating tissue has been observed. Interestingly, 16 papers mention that the lack of haptic feedback during surgery can be considered as a deficit. Simultaneously, several authors state that visual feedback can compensate for the lack of tactile feedback when operating on fragile tissue. While surgical robots are nowadays a common occurrence many hospitals across the world, the cost of acquiring and maintaining a surgical robot has been a frequent point of concern. Subsequently, 13 papers argue the cost of surgical robots prohibit their use. Especially in plastic surgery, limited data exists on the clinical benefits of surgical robots over conventional surgical intervention that would justify the adaptation of surgical robotics in common practice. Only one study assessed long-term outcome between robotic-assisted surgery compared to conventional reconstructive surgery. They discovered that functional post-operative outcomes 3 months following robotic-assisted oropharyngeal reconstruction is superior to conventional surgery (*p* = 0.005) (25). Finally, 10 papers highlighted that the size of surgical robotics can proof problematic in smaller operating theatres. Most operating rooms are not designed to facilitate large robots, therefore surgical workflow can be impeded.

**Table 2:** Surgical robotics and interventions in patients.

	Number of patients	Percentage
Total number of patients	114	100
**SURGICAL ROBOT**		
da Vinci Surgical System^®^	94	82.5
Automated Endoscopic System for Optical Positioning^®^	20	17.5
**SURGICAL TREATMENT**		
Head and neck surgery		
Oropharynx repair radial forearm fasciocutaneous flap	40	35.1
Oropharynx repair anterolateral thigh flap	4	3.5
Oropharynx repair facial artery myomucosal flap	1	0.9
Oropharyngeal repair primary closure	1	0.9
Cleft palate reconstruction	10	8.8
Latissimus dorsi harvest	2	1.8
Breast surgery		
Internal mammary vessel harvest	20	17.5
Latissimus dorsi harvest	6	5.3
Upper limb surgery		
Peripheral nerve translocation	7	6.1
Peripheral nerve repair	4	3.5
Ulnar artery reconstruction	1	0.9
Lower limb surgery		
Rectus muscle harvest	5	4.4
Peripheral nerve repair	2	1.8
Pelvic surgery		
Rectus muscle harvest	6	5.3
Brachial plexus surgery		
Brachial plexus reconstruction	5	4.4

## 4. Discussion

Following a variety of proof of concept studies in animal models and human cadavers, significant success has been documented in utilising surgical robots in patients within plastic surgery. There is a wide range of surgical procedures in which robotic surgery is feasible, including head and neck reconstruction, free-flap harvest and nerve reconstruction (Table 2). Improved visualisation of the operating field, enhanced dexterity and elimination of tremor are valuable aids when performing complex procedures on microscopic level. Consequently, the potential of surgical robotics is promising. Despite positive results from feasibility studies, uptake of surgical robotics within plastic surgery has been limited. The use of surgical robotics in day-to-day practice is restricted to a small subset of procedures, which is congruent with currently available literature. The majority of studies considered in this review are case reports or small case series with less than five patients. These provide valuable insight into important aspects of plastic surgery, such as microvascular anastomosis and nerve repair that form the basis of complex reconstruction. Although this may act as a stepping stone to conduct further research into surgical robotics in reconstructive microsurgery on a larger scale, this has not yielded in publication of larger studies. Yet, one area within the field of plastic surgery is currently leading the game when it comes to utilising surgical robots in common practice. Robotic head and neck reconstruction has seen remarkable progress over the last few years. Transoral robotic surgery is currently gaining popularity and is providing new approaches to oropharyngeal access in resection of cancers and cleft palate reconstruction. To date, results from transoral surgery have been promising, and importantly, data indicating this has been derived from comparative cohort studies (15, 25). Biron et al. compared transoral robotic surgery (*n* = 18) to the lip-splitting mandibulotomy approach (*n* = 29) for primary resection of oropharyngeal carcinomas with radial forearm free-flap reconstruction. Patients who underwent robotic resection and reconstruction were discharged from hospital approximately 5.3 days earlier, and estimated overall cost of admission was lower ($18,522.18 vs $24,932.16) compared to conventional surgery (15). Analysis of functional outcome by Tsai et al. between robotic-assisted free-flap oropharyngeal reconstruction (*n* = 14) and conventional free-flap reconstruction (*n* = 33) revealed that Functional Intraoral Glasgow Scale (47) was significantly better 3 months post-operatively in patients who underwent robotic reconstruction (*p* = 0.005) (25). As the indications for surgical robots are gradually increasing, the demand for adequate training and obtaining hands-on experience is rising. As such, the level of interest in this field is emerging and driven the creation of professional bodies, such as the Robotic-Assisted Microsurgical & Endoscopic Society (RAMES), to provide surgeons with training opportunities with robotic technology. Nevertheless, while increasing awareness of surgical robots through educational events encourages adaption of such systems on a larger scale, the future of surgical robots mainly will rely on the outcome of larger clinical studies. The availability of strong evidence from large studies is essential in order to determine if there is an overall benefit of surgical robotics over conventional microsurgery. A lack of meaningful evidence is currently the main barrier in order to assess whether surgical robots should be incorporated within plastic surgery on a larger scale.

### 4.1. Limitations of Surgical Robotics

#### 4.1.1. Instrumentation

A significant proportion of studies that were assessed in this review state that the absence of adequate instrumentation specifically designed for microsurgery poses a major limitation of current robotic surgical systems. The majority of published articles relied on the da Vinci surgical robot for assessing the feasibility of robotic microsurgery. While this particular system is licensed for minimally invasive surgery in seven surgical specialties, it is neither indicated nor designed for open plastic reconstructive microsurgery. The majority of instruments compatible with the da Vinci are considered to be too large for fine manipulation of delicate tissue normally seen during microsurgery. Success has been observed by utilising the da Vinci’s Black Diamond Micro Forceps for operating on small vessels and nerves (14, 20, 25, 36). Yet, lack of a comprehensive set of appropriate microsurgical instruments means that handling submillimetre tissue and equipment is challenging and time-consuming process. Common elements of a microsurgical procedure, such as dissecting blood vessels, applying vessel clamps and handling fine sutures, become more difficult when using a surgical robot compared to conventional microsurgery. In addition to oversized instruments, the variety of instruments of surgical tools does not cover the full scope of tissue encountered during microsurgery. Numerous operations involving upper or lower limbs involve manipulating a variety of tissue with different characteristics, such as skin, vessels and bones. To date, no robotic system exists that provides the tools necessary to perform procedures in which all different types of tissue are involved. As a result, performing procedures that involve both soft and hard tissues cannot be achieved purely using surgical robotics (12). In addition, the extent of macroscopic and microscopic techniques used during reconstructive surgery make robotic use inefficient. Consequently, it requires continuous switching between conventional and robotic-assisted microsurgery, which is labourious and a time-consuming process. 

Besides adequate surgical tools to operate on delicate tissue, appropriate optical aids to magnify and improve visualisation of the surgical field are essential during microsurgery. The da Vinci surgical robot provides endoscopic 3D imaging system capable of up to 10× magnification through digital zoom. Unfortunately, the image magnification and quality of da Vinci surgical robot is below standards compared to that of surgical microscopes (23). As microsurgical procedures occasionally may require magnification beyond what is currently offered with the da Vinci, this systemis limited in its use. Integration of surgical microscopes or other imaging systems capable of delivering adequate optical magnification while maintaining high image quality isdesirable. Overall, the scarcity of adequate microsurgical instruments and robotic systems tailored to the requirements of plastic and reconstructive microsurgery prohibits using the theoretical potential of surgical robots.

#### 4.1.2. Tactile Feedback

A recurring aspect of surgical robotics which some clinicians have argued as a disadvantage is the lack of tactile feedback when operating. A substantial proportion of papers reviewed, mention the absence of haptic feedback in current surgical robots as a drawback (*n* = 17). However, whether this is a disadvantage is controversial. Despite criticising the lack of haptic feedback, several go on to mention that tactile feedback is non-essential and can be compensated for by other means (11, 12, 28). While being able to sense the amount of forces exerted on delicate tissue may at first glance seem essential, it has been shown that visual feedback during microsurgery can reliably compensate for this deficit. In addition, some argue that the forces exerted during microsurgery are too low for humans to experience and therefore not appropriate to rely on. Despite data indicating that tactile feedback is non-essential, having such feature on surgical robots can provide benefits. Although soft tissue can show deformity during manipulation, this is not the case for rigid instruments used during the procedure. Several clinical studies noted that the lack of tactile feedback can result in bending needles, particularly when handling the needle using two robotic arms (19, 31, 43, 44). Furthermore, tactile feedback may become useful when implemented such that forces exerted are scaled on the surgeon’s end. Artificially increased forces such that a surgeon can feel and judge these could potentially reduce any unnecessary trauma to delicate tissue. Nevertheless, whether the availability of tactile feedback can reduce the risk of soft-tissue damage needs to be thoroughly validated. Future studies may provide a better insight into tissue trauma between conventional manual microsurgery and robotic microsurgery with tactile feedback.

#### 4.1.3. Cost

High costs of buying, using and maintaining surgical robotic systems have been a recurring theme throughout the literature. The cost of a single surgical robot can be in excess of $2 million (48), hence investing in surgical robots requires significant resources. Further direct and indirect costs arise that are essential to operate the system and provide a safe environment for robot-assisted surgery. Consumables required during a procedure can cost between $1,800 and $4,600 per instrument (49). Resources have to be allocated to provide training for operating theatre staff in order to familiarise them with surgical robots. Outside the operating room, further staffing is needed to reassure that these systems work reliably. Surgical robots are inherently complex systems that require specific expertise for repair and maintenance. Hence, hospitals that utilise surgical robots are required to negotiate service contracts with manufacturers that cost approximately an additional 10% of the system annually (49, 50). The spiralling costs associated with increased demands in staffing and consumables make the use of surgical robots less attractive. 

Offering expensive treatment options can be favourable for hospitals if these are associated with improved outcome or increased income in long term. However, a little data exists that suggests that this is indeed the case for plastic reconstructive microsurgery. Right now, few arguments exist to justify investing significant resources into surgical robots. In addition, published data indicates that robotic-assisted microsurgery is associated with prolonged operating time compared to conventional microsurgery (9, 10, 15, 22, 34, 42). Subsequently, the number of patients that can be treated may decrease and waiting times suffer. Despite the claimed paradoxical cost reduction of surgical robots related to shorter hospitalisation and post-operative complications (15, 48), the overall benefits do not yet outweigh the capital required to justify its use in plastic and reconstructive microsurgery. Without an increased turnover of patients and improved cost-efficiency, very few plastic surgery departments will be willing to invest in robotic-assisted surgery at the moment.

#### 4.1.4. Education

Surgical training often involves an apprenticeship model in which trainees initially observe a qualified professional and slowly gain skills through increasing their involvement during procedures. Surgical robots are often limited to a single surgeon controlling an entire system and therefore solely performs the whole procedure. As a result, there is little opportunity for assistants to get involved during robot-assisted operations. A lack of active involvement of surgeons may restrict their exposure to surgical robotics and limits opportunities to advance their skill set. There are two possible solutions to this dilemma, namely switch between surgeons during a procedure or make use of two or more full surgical robotic systems. While switching between different users is a relatively quick procedure, it is time consuming and delays may pose a threat to clinical outcomes during critical situations. Using multiple surgical robots may be a safer option during training young surgeons. This provides the lead surgeon with an assistant who can aid during the procedure, as well as allows trainees to gain skills necessary for robotic microsurgery. However, a single robotic system can cost in excess of $2 million, buying such system purely for training purposes is difficult to justify. Finally, it is important to appreciate that surgical robots should be considered as an additional tool, not as a replacement for conventional microsurgery. There are notable differences between the skills required for either form of microsurgery. Movements and handling of delicate tissue are significantly different, hence skills to successfully operate in such circumstances using a surgical robot are not directly transferable to conventional microsurgery. Future surgeons will need to be trained in both conventional and robotic-assisted microsurgery techniques to be capable of dealing with the wide variety of surgical problems that they will encounter throughout their career. Therefore, both robotic-assisted and conventional microsurgical experience should be incorporated into surgical training.

#### 4.1.5. Surgical Workflow

Studies have suggested that in certain scenarios using surgical robots is less convenient compared to conventional manual microsurgery. Willems et al. showed that during microsurgery with sufficient access to the surgical field, conventional surgery requires less operating time compared to robotic-assisted microsurgery (45). Reviewing patients and planning procedures in advance to determine an optimal treatment plan is essential before any intervention. Nevertheless, a degree of uncertainty will always exists and predicting which operations offer good surgical access and which do not may be difficult. Consequently, there is a risk that surgeons may have to switch between robotic and conventional surgery during a procedure to achieve a desired result. Transitioning during a procedure is by all means possible, though it is a labourious and time-consuming process that depends on the familiarity of operating room staff with surgical robots. Any delays as a consequence of this process may lead to increased costs. Furthermore, any event that prolongs operating and anaesthetic time could increase the risk of complications. To optimise surgical workflow, surgical robots must account for uncertainty during microsurgery and need to be able to provide a smooth and quick transition between conventional and robotic microsurgery.

## 5. Conclusion

Criticising technology based on what it is not capable of doing and pointing out flaws may be easy. Yet, highlighting such deficiencies and reflecting on what aspects could be improved is an essential tool to advancing its practice. Numerous research has highlighted that surgical robots can offer substantial benefits when operating. Surgical robots allow surgeons to perform extremely fine manoeuvres within narrow spaces that are near impossible during conventional surgery. Despite numerous advances of surgical robotics and the associated improved clinical outcomes seen in several surgical specialties, the robotic systems that are currently available do not offer overall benefits over conventional reconstructive microsurgery. On one hand, current systems are not designed to capture the full scope of complex reconstructive surgical procedures. While at the same time, microsurgical procedures themselves are not designed to make use of the full potential of surgical robots. Instead of trying to adapt surgical robots to current practice, more success may be achieved by designing microsurgical procedures around surgical robots. Surgical robots offer theoretical benefits that could make complex microsurgery more convenient, yet there are significant barriers that have prevented integrating surgical robotics in common practice. To date, no systems exist that are specifically designed for plastic and reconstructive microsurgery. Without adequate microsurgical instrumentation and training, surgeons will be hesitant to adapt robotic systems in their operating theatre. Current microsurgical procedures are not designed to make use of the full potential of surgical robots. A lack of strong quantitive data can be considered as a limitation of this review, there is nevertheless strong evidence that, without the deficiencies mentioned being resolved, little change can be expected within the foreseeable future.

## Author Contributions

YT: review design, data collection, data analysis and wrote the manuscript. PL: clinical advisor and data collection. JW: study supervision, review design and data interpretation. All authors reviewed and edited the manuscript.

## Conflict of Interest Statement

The authors declare that the research was conducted in the absence of any commercial or financial relationships that could be construed as a potential conflict of interest.
